# Residual tumor model in esophageal squamous cell carcinoma after neoadjuvant immunochemotherapy: Frequently involves the mucosa and/or submucosa

**DOI:** 10.3389/fimmu.2022.1008681

**Published:** 2022-12-09

**Authors:** Lei Gao, Zhi-Nuan Hong, Long Wu, Yinghong Yang, Mingqiang Kang

**Affiliations:** ^1^ Department of Thoracic Surgery, Fujian Medical University Union Hospital, Fuzhou, China; ^2^ Key Laboratory of Cardio-Thoracic Surgery (Fujian Medical University), Fujian Province University, Fuzhou, China; ^3^ Key Laboratory of Ministry of Education for Gastrointestinal Cancer, Fujian Medical University, Fuzhou, China; ^4^ Fujian Key Laboratory of Tumor Microbiology, Fujian Medical University, Fuzhou, China; ^5^ Department of Pathology, Fujian Medical University Union Hospital, Fuzhou, China

**Keywords:** neoadjuvant immunochemotherapy, residual tumor, esophageal squamous cell carcinoma, regression model, wait and see

## Abstract

**Objectives:**

The efficacy and safety of neoadjuvant immunochemotherapy (nICT) are widely explored in locally advanced esophageal squamous cell carcinoma (ESCC). Whether the “wait-and-see” strategy is applicable in ESCC after nICT is still lacking a theoretical basis. This study aimed to preliminarily explore the distribution of residual tumors and the regression pattern of ESCC after nICT.

**Methods:**

Patients undergoing radical esophagectomy after nICT in Fujian Medical University Union Hospital between January 2020 and March 2022 were identified. The resection specimens were re-evaluated by one experienced pathologist. The pathological response was assessed by tumor regression grade (TRG) (modified Ryan scheme). The TRG grade was divided into grades 0 (pathological complete response), 1, 2, and 3. The pathological stage was evaluated in the Eighth Edition AJCC. In the non-pCR group, the residual model was divided into four types: Type I, regression towards the lumen; type II, regression towards the invasive front; type III, concentric regression; and type IV, scattered regression.

**Results:**

A total of 95 consecutive patients were included for analysis. Seventy-six (80.0%) of 95 patients were in non-pCR (pathological complete response), and nine patients (9/76, 11.84%) had isolated residual tumors in lymph nodes. There was no significant difference in baseline characteristics between the pCR group and the non-pCR group (*p* > 0.05). The overall distribution of TRG for all esophageal wall layers was TRG 0 = 28 (28/95, 29.5%), TRG 1 = 17 (17/95, 17.9%), TRG 2 = 18 (18.9%, 18/95), and TRG 3 = 32 (32/95, 33.7%). In 67 patients with residual tumors in the esophageal wall (TRG ≧1), 63 (63/67, 94.0%) had residual tumor cells in the mucosa and/or submucosa, and four had isolated residual tumors in the muscle layer (4/67, 6.0%). Further analysis showed eight (8/67, 11.9%) patients with submucosal involvement but without mucosal involvement. The distribution of regression patterns was type I (*n* = 35, 52.2%), type II (*n* = 3, 4.5%), type III (*n* = 8, 11.9%), and type IV (*n* = 21, 31.3%).

**Conclusions:**

The mucosa and/or submucosa are frequently involved in residual malignancy, and the frequent regression models are regression toward the lumen and random regression. There is an opportunity to carefully test the residual tumors in a subgroup of the population with ESCC following nICT. However, some patients had residual tumors only in the muscle layer or lymph nodes. The clinical application of the wait-and-see strategy in ESCC after nICT should be explored using an appropriate evaluation protocol.

## Background

About 50% of newly diagnosed esophageal cancer (EC) occurs in China, and among them, more than 90% of patients are diagnosed with esophageal squamous cell carcinoma (ESCC) (1, 2). The neoadjuvant therapy following radical esophagectomy has been proven to improve long-term survival in ESCC (3–5). However, the standard neoadjuvant therapy for locally advanced ESCC is still uncertain. Neoadjuvant chemoradiotherapy (nCRT) is widely used in western countries, while neoadjuvant chemotherapy (nCT) is widely used in China and Japan. Due to the high recurrence rate, the long-term survival of ESCC after neoadjuvant therapy is still challenging. The 10-year results of the CROSS trial showed that the disease-free survival in the nCRT group is 63.6%, and the rate of distant metastasis is 24.3% (6). A more potent systemic therapy is urgently needed to improve long-term survival.

The feasibility and safety of adjuvant immunotherapy have been confirmed (7, 8). We previously conducted two single-arm phase II clinical trials to preliminarily confirm the safety and efficacy of neoadjuvant chemoimmunotherapy (nICT) in ESCC (9, 10). Recently, one multicenter clinical trial reported that the pathological complete response (pCR) was identified in 20 (39.2%) patients, and about 34 patients (56.7%) had three to four treatment-related adverse events (TRAEs) (11). One meta-analysis (including 20 studies and 621 patients) found that the pCR was 33.8% (95% CI: 29.6%–37.9%) and grades 3–4 TRAE rate was 19.4% (95% CI: 11.5%–31.5%) in EC after nICT (12). The nICT pattern is promising and has the potential to be the standard treatment for locally advanced ESCC. Based on the promising short-term results in phase II clinical trials, some phase III clinical trials are being conducted to further confirm the efficacy of nICT in locally advanced ESCC (such as NCT05043688) (13).

Considering the trauma of esophagectomy, patients who respond completely to nICT may benefit from the “wait-and-see” strategy (14). Endoscopy with bite-on-bite biopsies is one of the most important tools during active surveillance. Thus, the distribution of residual tumors and the regression model of the primary tumor are of great importance in detecting residual tumors. Currently, there are still no data reporting the features of residual tumors after nICT. Thus, this study aimed to preliminarily explore the distribution of residual tumor and regression pattern of ESCC after nICT.

## Methods

### Patient selection and study design

This is a retrospective study based on prospectively collected data. We included consecutive patients who underwent radical esophagectomy after nICT for ESCC at Fujian Medical University Union Hospital Thoracic Department from January 2020 to March 2022. The inclusion criteria included the following: diagnosis of locally advanced ESCC before treatment (clinical staged with cT1N1-3M0 or cT2-4aN0-3M0), thoracic ESCC, and undergoing radical esophagectomy. The exclusion criteria included the following: patients diagnosed with other pathological types, patients with a history of the malignant tumor within 5 years, patients receiving surgery alone, patients with other neoadjuvant therapies (including nCRT, nCT, or combinations with other target therapies), and patients who underwent exploratory surgery or palliative surgery.

### Treatment protocol

The pretreatment clinical stage was evaluated by enhanced computed tomography (CT), positron emission tomography-computed tomography (PET-CT), and ultrasound. Based on the thoracic-abdominal enhanced CT or ultrasound, patients with at least two of the following characteristics: round shape, nonhomogeneous density, and short axis ≥ 10 mm were considered clinical node-positive (cN+). The cN+ should also be considered when PET-CT suggests a high intake of fluorodeoxyglucose. The tumor was staged using the Eighth Edition American Joint Committee on Cancer/Union for International Cancer Control staging system (AJCC).

We have already conducted two phase II clinical trials (ChiCTR2100052784 and ChiCTR2100045659). Patients with locally advanced ESCC were given two to four cycles of PD-1 inhibitors (including sintilimab at 200 mg, toripalimab at 240 mg, pembrolizumab at 200 mg, and camrelizumab at 200 mg) in a combination of neoadjuvant chemotherapy every 3 weeks. Previous reports (9, 10) provided details on nICT. Within 4–6 weeks after neoadjuvant therapy, the patient underwent another chest and upper abdominal CT or PET-CT scan for clinical evaluation. If there is no metastatic disease or the tumor progresses to being unresectable, radical resection of esophageal carcinoma is performed. Patients received thoracoscopic or robot-assisted McKeown minimally invasive esophagectomy (MIE), with a thoracotomy if necessary. We routinely performed two-field lymphadenectomy, and a standard three-field lymphadenectomy was performed when preoperative evaluation suggested cervical lymph node metastasis.

### Pathological evaluation

All patient specimens were systematically reassessed by an experienced specialist in Fujian Medical University Union Hospital (Long Wu) and confirmed by other pathologists when there were uncertain slides. The evaluation focused on the depth of pretreatment invasion, resection margins, response to the primary lesion, and metastatic lymph nodes prior to treatment. The primary outcomes were the distribution of residual tumor and the regression pattern of ESCC after nICT. The pathological TNM stage was staged using the Eighth Edition AJCC.

**Figure 1:**
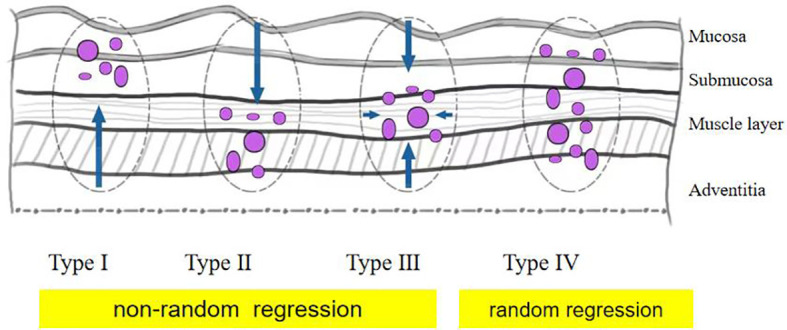
Schematic diagram of tumor regression pattern including type I, type II, type III, and type IV.

**Figure 2:**
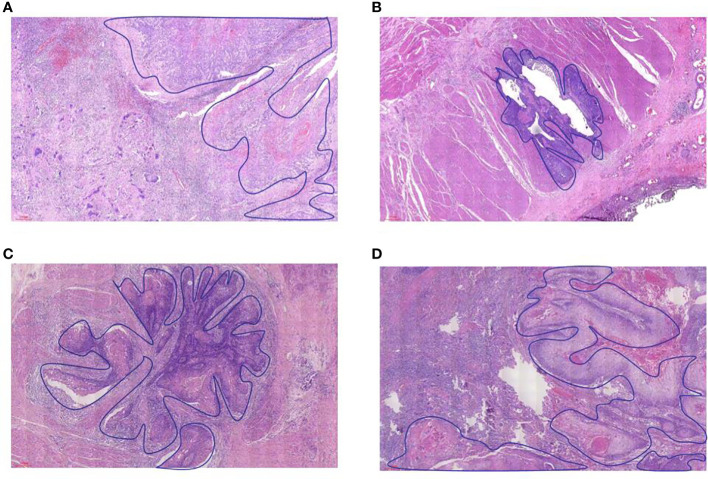
The example of residual tumor and regression model in the esophageal wall. **(A)** type I, **(B)** type II, **(C)** type III, and **(D)** type IV.

The pathological response was assessed by tumor regression grade (TRG) (modified Ryan scheme). The TRG grade was divided into grade 0 (complete pathological response in both primary and lymph nodes), grade 1 (near-complete response), grade 2 (partial response), and grade 3 (poor or no response) (15, 16). In the non-pCR group, the residual model in the esophageal wall was classified into four types: type I: regression towards the lumen with more residual tumors in the mucosa and submucosa; type II: regression towards the invasive front with more residual tumors in the muscular propria and adventitia/surrounding stroma; type III: concentric regression with more residual tumors in the submucosa and muscularis propria; and type IV: scattered regression with a comparable amount of residual tumors in all layers). Types I, II, and III were defined as nonrandom regression groups, and type IV was defined as a random regression group. The regression model is summarized in **Figure 1** (17, 18). The example of the regression model is summarized in **Figure 2**.

### Statistical analysis

The continuous variables with abnormal distribution were represented by the median (interquartile range), and the continuous variables with normal distribution were represented by mean (standard deviation (SD)). Classification variables were expressed in numbers (percentage). For equivalent variables with normal distribution, an independent Student’s *t*-test was used. Mann–Whitney *U* test was used to compare the abnormal distribution variables between the two groups. The frequency of categorical variables was determined by the Chi-square test or Fisher’s exact test. R version 4.0.4 (r foundation for statistical computing, Vienna, Austria) was used for statistical analysis. A *p*-value of < 0.05 indicated a significant difference.

## Results

### Patient selection and baseline characteristics

**Figure 3:**
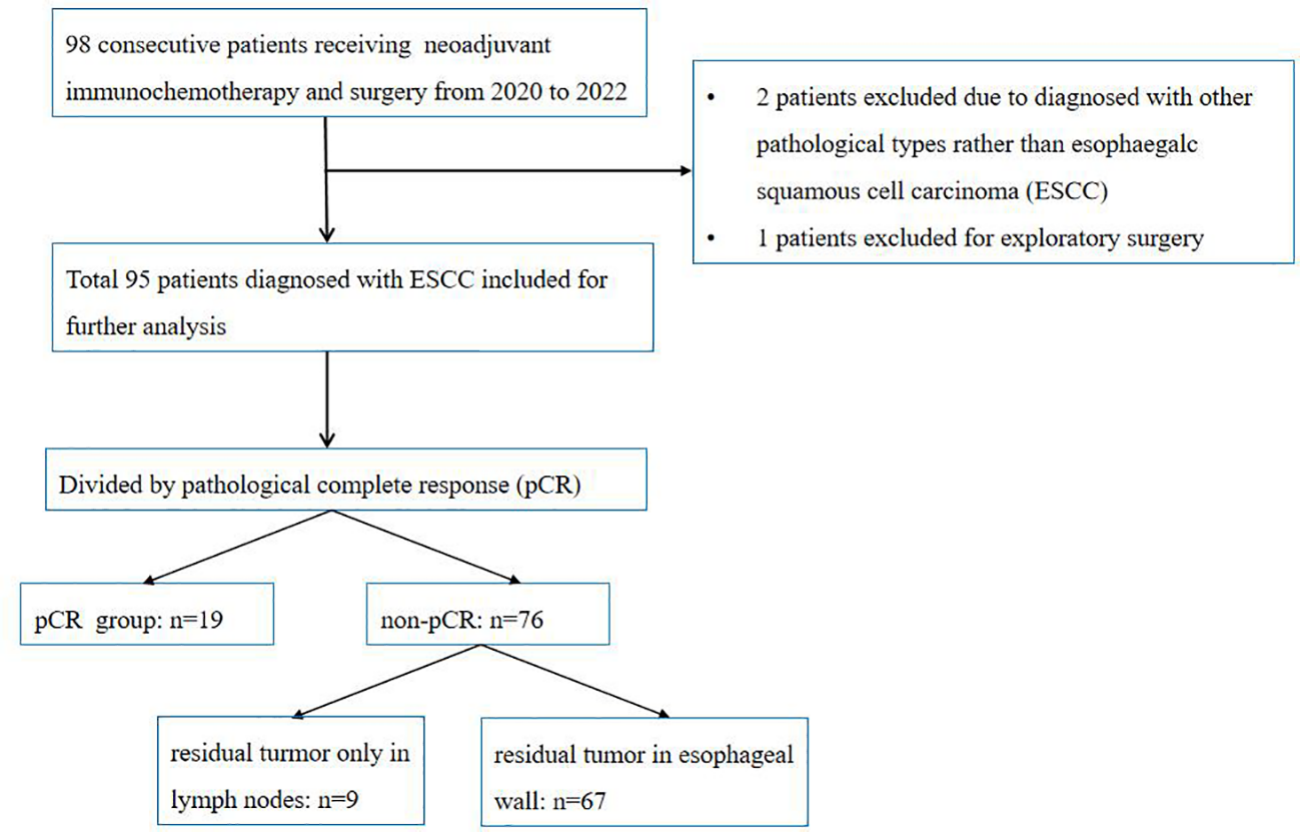
Patient selection flowchart.

The patient selection chart details are summarized in **Figure 3**. Two patients were excluded due to being diagnosed with other pathological types rather than ESCC, and one patient was excluded due to exploratory surgery. Finally, a total of 95 patients diagnosed with ESCC were identified for further analysis, including 23 women and 72 men. The median age was 60.45 ± 6.75 years old. The tumor location was distributed as follows: nine (9.47%) in the upper third, 49 (51.58%) in the middle third, and 37 (38.95%) in the lower third. The median interval to surgery was 42 days (34, 50 days). The median number of resected lymph node number was 36 (29,42).

There was no significant difference in sex, age, body mass index (BMI), tumor location, preclinical T stage, and preclinical N stage (*p* > 0.05). The median interval to surgery was 42 and 41 days in the non-pCR group and pCR group, respectively. The median number of resected lymph nodes was 36 in both the non-pCR group and the pCR group (*p* > 0.05). Comparisons of baseline characteristics between the pCR group and the non-pCR group are summarized in **Table 1**.

**Table 1:** Comparisons of baseline characteristics between the pCR group and the non-pCR group.

Variables	Total (*n* = 95)	Non-pCR group (*n* = 76)	pCR group (*n* = 19)	*p*-value
Tumor location (*n* (%))				
Upper third	9 (9.47)	7 (9.21)	2 (10.53)	0.76
Middle third	49 (51.58)	38 (50.00)	11 (57.90)	
Lower third	37 (38.95)	31 (40.79)	6 (31.58)	
Hypertension (*n* (%))				
No	77 (81.05)	63 (82.90)	14 (73.68)	0.36
Yes	18 (18.95)	13 (17.11)	5 (26.32)	
Drinking history (*n* (%))				
No	63 (66.32)	50 (65.79)	13 (68.42)	0.83
Yes	32 (33.68)	26 (34.21)	6 (31.58)	
Smoking history (*n* (%))				
No	41 (43.16)	33 (43.42)	8 (42.11)	0.92
Yes	54 (56.84)	43 (56.58)	11 (57.90)	
Diabetes				
No	89 (93.68)	71 (93.42)	18 (94.74)	0.83
Yes	6 (6.32)	5 (6.58)	1 (5.26)	
Preclinical N stage (*n* (%))				
cN0	29 (30.53)	25 (32.90)	4 (21.05)	0.32
cN1-3	66 (69.47)	51 (67.11)	15 (78.95)	
Preclinical T stage (*n* (%))				
cT1-2	18 (18.95)	14 (18.42)	4 (21.05)	0.79
cT3-4	77 (81.05)	62 (81.58)	15 (78.95)	
Sex (*n* (%))				
Female	23 (24.21)	18 (23.68)	5 (26.32)	0.81
Male	72 (75.79)	58 (76.32)	14 (73.68)	
Body mass index (median [IQR])	21.48 [20.37, 22.77]	21.67 [20.37, 22.84]	21.22 [20.20, 22.67]	0.60
Albumin (g/L) (mean ( ± SD))	41.91 ( ± 3.66)	41.96 ( ± 3.75)	41.68 ( ± 3.27)	0.77
Time to surgery (days) (median [IQR])	42 [34, 50]	42 [34, 52]	41 [34, 44]	0.47
Lymph nodes moved number (median [IQR])	36 [29, 42]	36 [28, 43]	36 [31, 40]	0.99
Age (years) (mean ( ± SD))	60.453 ( ± 6.718)	60.434 ( ± 6.420)	60.526 ( ± 7.796)	0.96

pCR, pathological complete response.

### Distribution of residual tumors in the esophageal wall and lymph nodes in ESCC after nICT

The details of the pathological response are summarized in **Table 2**. The overall distribution of TRG regression for all esophageal wall layers was TRG 0 = 28, TRG 1 = 17, TRG 2 = 18, and TRG 3 = 32. In total, there were 19 pCR (ypT0N0) patients and 76 non-pCR. Among the non-pCR group, a total of nine patients were ypTON+, of whom five were diagnosed with pre-cT3–4. In other words, nine patients had residual tumors only in their lymph nodes. In the subgroup of pretreatment cT3–4 (*n* = 77), the overall distribution of TRG regression was TRG 0 = 20, TRG 1 = 15, TRG 2 = 15, and TRG 3 = 27. TRG regression was distributed as TRG 0 = 58, TRG 1 = 4, TRG 2 = 9, and TRG 3 = 24 in lymph nodes.

**Table 2:** The details of pathological response in esophageal squamous cell carcinoma after neoadjuvant immunochemotherapy.

Contents	Variables	Number
ypT stage (*n* (%))	0	28 (29.47)
	1	21 (22.11)
	2	10 (10.53)
	3	36 (37.90)
ypN stage (*n* (%))	0	52 (54.74)
	1	33 (34.74)
	2	9 (9.47)
	3	1 (1.05)
ypTNM stage (*n* (%))	yPCR	19 (20.00)
	I	20 (21.05)
	II	15 (15.79)
	IIIa	19 (20.00)
	IIIb	21 (22.11)
	IVA	1 (1.05)
ypTNM model (*n* (%))	ypT0N0	19 (20.00%)
	ypT0N+	9 (9.47%)
	ypT+N0	33 (34.74%)
	ypT+N+	34 (35.79%)
Lymph node pathological evaluation (*n* (%))	TRG = 0	58 (61.05)
	TRG = 1	4 (4.21)
	TRG = 2	9 (9.47)
	TRG = 3	24 (25.26)
Pathological response in primary tumor (*n* (%))	TRG = 0	28 (29.47)
	TRG = 1	17 (17.90)
	TRG = 2	18 (18.95)
	TRG = 3	32 (33.68)
Regression model of primary tumor (*n* = 67) (*n* (%))	Type I	35 (52.24%)
	Type II	3 (4.48%)
	Type III	8 (11.94%)
	Type IV	21 (31.34%)
Regression model of primary tumor in pre-cT3–4 (*n* = 57) (*n* (%))	Type I	32 (56.14%)
	Type II	2 (3.51%)
	Type III	7 (12.28%)
	Type IV	16 (28.07%)

**Figure 4:**
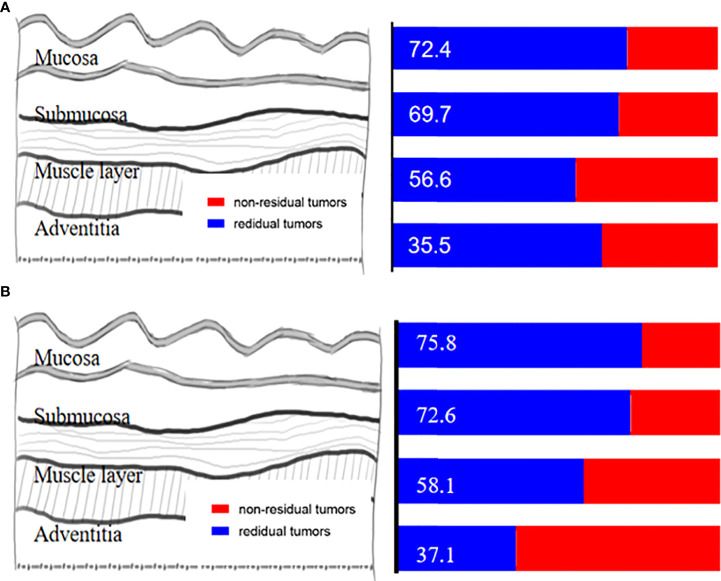
**(A)** The exact location of the remaining tumor in the esophageal wall. **(B)** The exact location of the remaining tumor in the esophageal wall among patients with pre-cT3-4.

In the non-pCR group (*n* = 76), the exact location of the remaining tumor was identified in the mucosa, submucosa, the muscle layer, adventitia, and lymph nodes in 72.4%, 69.7%,56.6%, 35.5%, and 56.6% of the cases, respectively (**Figure 4A**). Among patients with residual tumor in the esophageal wall (*n* = 67), only four (4/67, 6.0%) had no residual in both mucosa and submucosa, with only a residual tumor in the muscle layer, and eight (8/67, 11.9%) with submucosal involvement but without mucosal involvement. Further subgroup analysis among patients with pre-cT3–4 (*n* = 62) revealed that the exact location of the remaining tumor was identified in the mucosa, submucosa, muscle layer, adventitia, and lymph nodes in 75.8%, 72.6%,58.1%, 37.1%, and 54.8% of the cases, respectively (**Figure 4B**).

### Tumor regression pattern within the esophageal wall in ESCC after nICT

**Figure 5:**
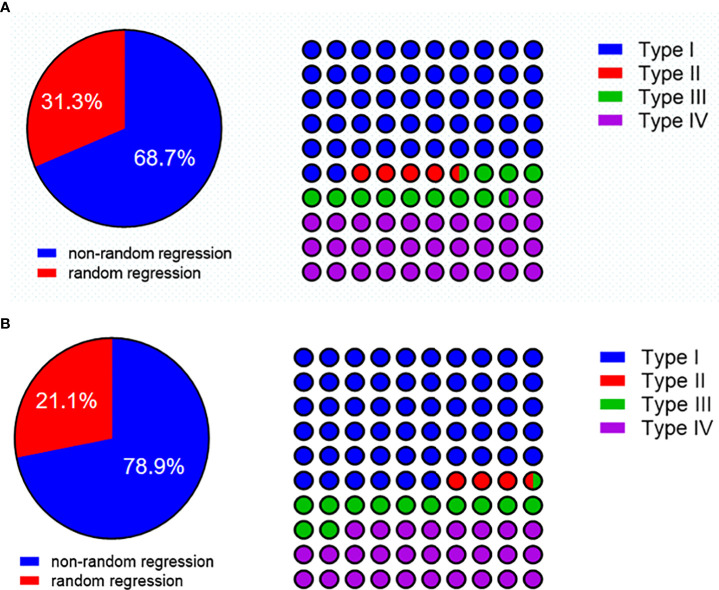
**(A)** The regression pattern in patients with residual tumors in the esophageal wall. **(B)** The regression pattern in pre-cT3-4 with a residual tumor in the esophageal wall.

Among patients with residual tumors in the esophageal wall (*n* = 67), a total of 46 (68.7%) had a nonrandom regression pattern and 21 (31.3%) had random regression. The distribution of regression patterns was type I (*n* = 35, 52.2%), type II (*n* = 3, 4.5%), type III (*n* = 8, 11.9%), and type IV (*n* = 21, 31.3%). Type I and type IV were the frequent regression models (**Figure 5A**).

To further characterize the tumor regression pattern, we did a subgroup analysis in pre-cT3–4 patients with residual tumors in the esophageal wall (*n* = 57). A total of 46 patients (71.9%) had a nonrandom regression pattern, and 16 (28.1%) had random regression. The distribution of regression patterns was type I (*n* = 32, 56.1%), type II (*n* = 2, 3.5%), type III (*n* = 7, 12.3%), and type IV (*n* = 16, 28.1%). Type I and type IV were still the frequent regression models (**Figure 5B**).

## Discussion

We found that 76 (80.0%) of 95 patients were non-pCR, and nine (9/76, 11.84%) had isolated residual tumors in lymph nodes. There was no significant difference in baseline characteristics between the pCR group and the non-pCR group (*p* > 0.05). In 67 patients with residual tumors in the esophageal wall (TRG ≧1), 64 (63/67, 94.0%) had residual tumor cells in the mucosa and/or submucosa, and four patients had isolated residual tumors in the muscle layer (4/67, 6.0%). Further analysis showed that eight (8/67, 11.9%) patients were with submucosal involvement but without mucosal involvement. Type I and type IV were the frequent tumor regression models. Patients diagnosed with pre-cT3–4 did not show any distinct tumor regression patterns.

Recently, a meta-analysis including phase II clinical trials indicated that ESCC patients after nICT had a median pCR rate of 33.8%. Considering the promising response, we conducted this study to evaluate whether it was feasible to introduce a wait-and-see strategy in ESCC patients following nICT. For patients with residual tumors, timely detection of residual viable tumor cells in a wait-and-see strategy is important. To make an accurate and appropriate detection, we need to know where and how many residual tumor cells remain. However, little was known about the characteristics of residual tumors in ESCC patients undergoing nICT. Previously, Shapiro et al. found that 31 (30%) of 102 EC patients were pCR, while 63 were non-pCR (63/71, 89%), and residual tumor cells were frequently observed in the mucosa and/or submucosa (17). Tang et al. found that a total of 115 ESCC patients (115/138, 83.3%) had residual tumors in the mucosa or submucosa (18) after surgery. Chao et al. examined the distribution of residual tumors at the primary tumor site in patients with ESCC achieving who achieved major pathological response (MPR) after nCRT and found that residual tumors were frequently identified in the submucosa (54/76, 71%) and the mucosa (44/76, 58%) (19). In this study, we found that 63 patients (63/67, 96.0%) had residual tumor cells in the mucosa and/or submucosa. This finding supports the possibility of carefully testing residual tumors in a subgroup of the ESCC population following nICT.

The next step is to determine the depth of the endoscopic biopsies. Results of the preSANO trial indicated that a residual tumor was observed in the resection specimen from 27 of 49 patients after nCRT, despite endoscopic biopsies being negative. Eighteen patients had a residual tumor in the mucosa layer, eight patients had residual tumors only in the submucosa layer, and one patient had a tumor only in the muscle layer (20). Chao et al. investigated the anatomical locations of residual tumors in ESCC patients after nCRT using false-negative endoscopic biopsies and found that only three (6.1%) patients had a residual tumor in the muscle layer or the adventitia without simultaneous involvement of mucosa or submucosa layers (21). Fujishima et al. also highlighted the difficulty of detecting residual tumors using conventional endoscopic biopsy in ESCC patients after nCRT (22). In this study, only four (4/67, 6.0%) patients had isolated residual tumors in the muscle layer. Meanwhile, eight (8/67, 11.9%) patients had submucosal involvement but no mucosal involvement. As a result, we recommend that submucosal biopsies should be performed to reduce the FN rate, especially for patients with clinically suspected residual tumors. Furthermore, more biopsy specimens are recommended to improve active surveillance whenever possible.

Clinical complete responders should be accurately identified before active surveillance strategies can be offered to these patients. Due to the limitations of endoscopy biopsy, patients who only had a residual tumor in the muscle layer or in lymph nodes need a more comprehensive examination. Previously, Eyck et al. conducted a meta-analysis to evaluate the accuracy of endoscopic biopsies, EUS, or PET-CT for detecting residual disease after nCRT for EC and concluded that endoscopic biopsies, EUS, and 18F-FDG PET(-CT) as single modalities were insufficient (23). The preSANO trials concluded that endoscopic ultrasonography, bite-to-bite biopsy, and fine-needle aspiration of suspected lymph nodes were adequate for the evaluation of local residual lesions, with PET-CT for the detection of interval metastases (24). Recently, Wang et al. found that the parameters of 18F-FDG PET/CT (including maximum standardized uptake value (SUVmax), mean standardized uptake value (SUVmean), tumor-to-blood pool SUVmax ratio (SUVTBR), total lesion glycolysis (TLG), and metabolic tumor volume (MTV)) in scan-2 (prior to surgery) had an excellent predictive ability for the pCR of primary tumors. Furthermore, SUVmax in scan-2 had a high negative predictive ability (98.6%) with a cutoff value of 1.4 (25). There were no studies focusing on the application of endoscopic biopsies or EUS in ESCC after nICT. Further investigation is necessary to determine whether the preSANO evaluation protocol can be satisfied in ESCC after nICT.

## Limitations

**Figure 6:**
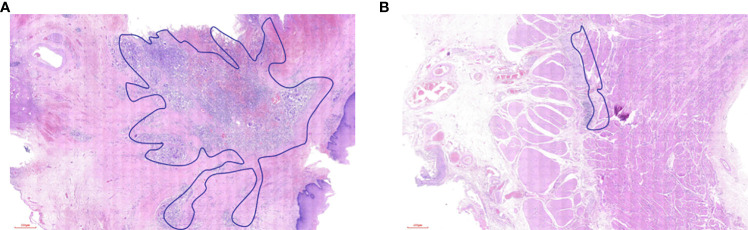
**(A)** The treatment response in the esophageal wall after neoadjuvant chemoradiotherapy. **(B)** The treatment response in the esophageal wall after neoadjuvant chemoimmunotherapy.

To the best of our knowledge, this was the first study to investigate the distribution of residual tumor and the regression pattern of ESCC after nICT. This study had the following limitations: Firstly, pathological sections were evaluated by a single pathologist to arrive at conclusions, which might limit the repeatability of the findings. To avoid potential bias, the pathological evaluation criteria used in this study were consistent (modified Ryan scheme). When evaluating the pathological response of the primary tumor, we did not evaluate the TRG per individual wall layer. Instead, we gave the overall TRG evaluation and the regression pattern of the primary tumor. Secondly, the TRG system was based on the ratio of residual tumor area to residual fibrotic area. Due to technical limitations, this ratio did not include the absolute area. Thirdly, the sample size was relatively limited and came from only a single institution. Meanwhile, due to the scarcity of patients with esophageal adenocarcinoma (EAC) in China, whether our findings were applicable to patients with EAC following nICT should be confirmed. Fourth, Tang et al. reviewed the postoperative pathology to predict cT staging (18), and we attempted to predict cT staging using this method as well. The histopathological features of tumor cell regression, necrosis with surrounding foam cell bands, granulation tissue formation, and peripheral fibrous tissue scarring in the original tumor area can all be used as evidence of tumor regression after treatment. The treatment response was obvious in patients with pCR after nCRT (**Figure 6A**). However, we noticed that the therapy response after nICT was not obvious, especially in patients with pCR (**Figure 6B**), which made predicting the cT stage difficult. Furthermore, with the prolongation of the interval to surgery, the therapy response would decrease. Thus, considering that a majority of the patients included in this study were from clinical trials with relatively accurate cT stage, we used the pretreatment clinical stage in the medical record system.

## Conclusion

The pathological response of ESCC after nICT is promising. The mucosa and/or submucosa layer has frequent residual malignant involvement, and the frequent regression models are regression toward the lumen and random regression. There is an opportunity to carefully test (including submucosal biopsies) the residual tumors in a subgroup of the population with ESCC after nICT. However, some patients had residual tumors only in the muscle layer or in lymph nodes. The clinical practice of the wait-and-see strategy in ESCC after nICT should be explored based on an adequate evaluation protocol.

## Data availability statement

The original contributions presented in the study are included in the article/supplementary material. Further inquiries can be directed to the corresponding authors.

## Ethics statement

The studies involving human participants were reviewed and approved by Fujian Medical University Union Hospital. The patients/participants provided their written informed consent to participate in this study. Written informed consent was obtained from the individual(s) for the publication of any potentially identifiable images or data included in this article.

## Author contributions

Z-NH and MK designed the study. Long We and YY re-reviewed the slicements. Z-NH and LG drafted the manuscript. All authors contributed to the article and approved the submitted version.
